# The impact of long-term low-dose ionizing radiation on human health: risks and protective measures

**DOI:** 10.3389/fmed.2026.1628683

**Published:** 2026-01-29

**Authors:** Yue Zhang, Rongrong Li, Xiaoling Li, Ru Li, Tiankun Lu, Xiaohu Zhong, Zexuan Li, Haifang Zhang, Junfeng Wang

**Affiliations:** 1 Department of Clinical Laboratory, The Nuclear Industry 417 Hospital, Xi’an, China; 2 Department of Medical Imaging, The Lintong Rehabilitation and Convalescent Center, Xi’an, China; 3 Department of Clinical Laboratory, The Second Affiliated Hospital of Soochow University, Suzhou, China

**Keywords:** human health, ionizing radiation, low dose, low dose rate, systems

## Abstract

**Purpose:**

With the widespread application of ionizing radiation in medicine and other fields, the health impacts of long-term occupational exposure to low-dose ionizing radiation (LDIR) on the human body have garnered growing attention. Despite inconsistencies in the findings across various studies, the effects of LDIR on human health are undeniable. There is an urgent need to comprehensively examine the impacts of LDIR on the entire human health system, clearly delineate the key aspects of occupational protection, optimize the working environment, and raise the awareness of self-protection among workers exposed to ionizing radiation.

**Conclusion:**

By synthesizing existing research findings on LDIR, we summarized its effects on the human immune, hematopoietic, endocrine, circulatory, digestive, reproductive, respiratory, and urinary systems. Within the immune system, the thyroid gland is the organ most susceptible to LDIR-induced damage. Sufficient protective measures should be implemented regardless of the specific work setting. Additionally, the adverse impacts of LDIR on the hematopoietic and reproductive systems are particularly noteworthy and cannot be overlooked.

## 1 Introduction

In the era of rapid technological advancement, ionizing radiation is closely intertwined with human health. As electronic devices become increasingly prevalent, the application of ionizing radiation technology in the medical field has expanded significantly. For example, in clinical practice, LDIR is utilized in radiotherapy and medical imaging procedures. Clarifying the impact of LDIR on the human health system has thus become an urgent priority. Ionizing radiation, primarily derived from X-rays and gamma rays, can strip electrons from atoms or molecules, thereby damaging cellular and tissue structures (1). The United Nations Scientific Committee on the Effects of Atomic Radiation (UNSCEAR) defines LDIR as an effective dose of ≤ 100.00 mSv, with corresponding organ absorbed doses for X/*γ* rays typically below 100 mGy and a low dose rate of < 6 mSv/year (2).

The health effects of LDIR are conceptualized within a dualistic model established by the International Commission on Radiological Protection (ICRP), categorized into deterministic effects and stochastic effects (3). Deterministic effects exhibit a dose threshold, below which they do not occur, and their severity increases with the dose (4). This is a consequence of substantial cell loss or functional impairment in tissues and organs, manifesting clinically as conditions such as cataracts, hematopoietic suppression, and skin damage. In contrast, stochastic effects are presumed to have no safe threshold; their probability of occurrence, not severity, increases linearly with dose in a linear-no-threshold (LNT) relationship. These effects result from unrepaired or misrepaired DNA damage in a single cell (somatic or germline), potentially leading to cancer many years later or heritable genetic diseases in offspring. Consequently, the fundamental objectives of radiological protection are to prevent the occurrence of deterministic effects and to limit the probability of stochastic effects to the lowest level reasonably achievable (5).

The LNT model has long served as the cornerstone of international radiation protection policies, asserting that any increase in ionizing dose, no matter how minimal, results in a proportional elevation in cancer risk. Proponents contend that this straightforward and conservative assumption is validated by epidemiological data from atomic bomb survivors and occupational cohorts, which seem to support a linear dose–response relationship for solid cancers and leukemia at low to moderate doses. However, a growing body of radiobiological evidence has called into question the universal applicability of the LNT model. Studies on DNA repair, adaptive responses, and bystander signaling have shown that cells exposed to low-dose, low-dose-rate radiation can activate protective mechanisms that reduce mutational load, suggesting a non-linear, potentially threshold-dependent response at doses below ~100 mSv. Meanwhile, the “threshold” or stochastic-effect model posits that stochastic outcomes, such as cancer, only occur after a specific dose threshold is exceeded, reflecting the balance between damage induction and intrinsic defensive mechanisms. Recent mechanistic research has identified dose-rate-dependent thresholds for chromosomal aberrations and micronucleus formation, indicating that under chronic exposure conditions, stochastic effects may not manifest until doses surpass several tens of milligray. These findings have prompted calls for a revised risk framework that incorporates dose-rate and individual susceptibility factors, rather than a single linear extrapolation. The third major perspective is radiation hormesis, which proposes that low-level radiation can elicit beneficial biological effects, including enhanced immune surveillance and increased lifespan. Epidemiological analyses of post-Hiroshima/Nagasaki cohorts and low-dose occupational groups have reported reduced cancer incidence and mortality at doses around 10–50 mSv, findings that are consistent with hormetic dose–response curves.

Editorials and analyses published in recent years argue that the weight of current evidence favors hormesis over the LNT model for low-dose, low-dose-rate exposures, and they advocate for policy adjustments to ease overly restrictive dose limits. Despite these advancements, the debate remains unresolved. The National Council on Radiation Protection and Measurements (NCRP) continues to endorse the LNT model in its latest commentary, citing the absence of conclusive epidemiological evidence supporting a threshold or hormetic effect. On the contrary, several regulatory agencies and scientific organizations have called for systematic reviews of the emerging mechanistic and epidemiological evidence, suggesting that a hybrid model—incorporating a low-dose threshold alongside a potential hormetic region—may more accurately reflect biological reality. In summary, contemporary research emphasizes three key lines of evidence: (1) the traditional LNT model is supported by long-term epidemiological data on high-dose exposure but may overestimate risks at very low doses; (2) mechanistic studies have uncovered dose-rate-dependent thresholds for stochastic damage, challenging the concept of a zero threshold; and (3) hormesis data demonstrate that low-dose radiation can exert protective effects. The field is evolving toward a more nuanced, data-driven risk assessment approach that balances caution with emerging evidence of adaptive and beneficial biological responses.

In the field of radiobiology, the “priming dose” effect (Raper-Yonezawa effect) of ionizing radiation—specifically the adaptive response induced by pre-exposure to low doses—interacts profoundly with human immune regulation and radiological protection strategies (6). This phenomenon is thought to be mediated by the activation of complex intracellular signaling networks (e.g., NF-κB, p53 pathways), which initiate a cascade of responses that enhance genomic stability and stress resistance. These responses include the upregulation of DNA damage repair efficiency, increased activity of antioxidant enzymes, and modulation of cell cycle checkpoints (7). From the perspective of radiological protection, clarifying the radiation adaptive response induced by a priming dose and its associated immune activation mechanisms not only identifies potential targets for the development of novel radioprotective agents (e.g., agonists or immunomodulators) but also challenges the traditional linear-no-threshold model. Moreover, it carries substantial theoretical and practical significance for optimizing occupational exposure protection standards.

Studies on populations residing in high-background radiation areas (HBRAs), such as Yangjiang in Guangdong Province (China), Kerala (India), and Ramsar (Iran), have provided valuable natural observation models. These studies have revealed that despite prolonged exposure to high background radiation, the health effects on the local populations are complex. Some individuals may have undergone “pre-adaptation” of their cellular defense systems through intergenerational or lifelong exposure, resulting in enhanced genomic stability and more robust DNA damage response capabilities. This phenomenon directs our focus to the molecular basis underlying individual differences: polymorphisms in DNA repair genes. A wealth of studies have documented a direct correlation between polymorphisms in key DNA repair genes (e.g., ATM, TP53, and XRCC1) and the capacity to induce adaptive responses. Thus, an individual’s radiation sensitivity profile is fundamentally shaped by their unique genetic makeup, which not only dictates their inherent vulnerability in the absence of radiation exposure but also determines their potential to develop radiation tolerance via the radiation adaptive response (RAR) mechanism (8). In conclusion, from systemic immune regulation to molecular-level gene polymorphisms, an individual’s radiation response is a multi-level, highly individualized biological process, which has crucial guiding significance for future precise radiation medicine, occupational protection, and environmental radiation risk assessment.

Currently, most studies focus on the effects of LDIR on the function of individual organs (e.g., thyroid function), while few systematically address the systemic impacts of LDIR on the human body. Therefore, this review summarizes the effects of LDIR on eight major human body systems, aiming to provide radiation workers with a more comprehensive and intuitive understanding of their health status. In turn, this will enhance their protective awareness in daily work and facilitate the implementation of more effective self-protection measures.

## 2 Methods

### 2.1 Search strategy

A comprehensive literature search was conducted in PubMed and Web of Science databases, covering publications up to November 20, 2025. Following the initial analysis, the reference lists of the selected publications were also searched for additional relevant studies. The search keywords used in the electronic databases were “low doses of ionizing radiation AND (immune system/hematopoietic system/endocrine system/circulatory system/digestive system/reproductive system/respiratory system/urinary system).” No restrictions were imposed on the language or publication date of the retrieved studies.

### 2.2 Study selection

A multi-stage approach was adopted for the literature review process. Seven authors independently performed duplicate screening and removal using ZOTERO software. After initial screening based on titles and abstracts, full-text articles were evaluated to determine their compliance with the inclusion criteria. For selected publications where the English full text was unavailable, the corresponding authors were contacted to verify whether the studies met the eligibility criteria.

### 2.3 Inclusion and exclusion criteria

The inclusion criteria were as follows: (1) studies involving workers occupationally exposed to LDIR; and (2) studies focusing on the effects of LDIR on the function and development of the eight major human body systems. The exclusion criteria were as follows: (1) studies unrelated to the research topic; and (2) studies involving workers with no exposure to LDIR.

## 3 Effects of long-term LDIR on the human health system

**Figure 1:**
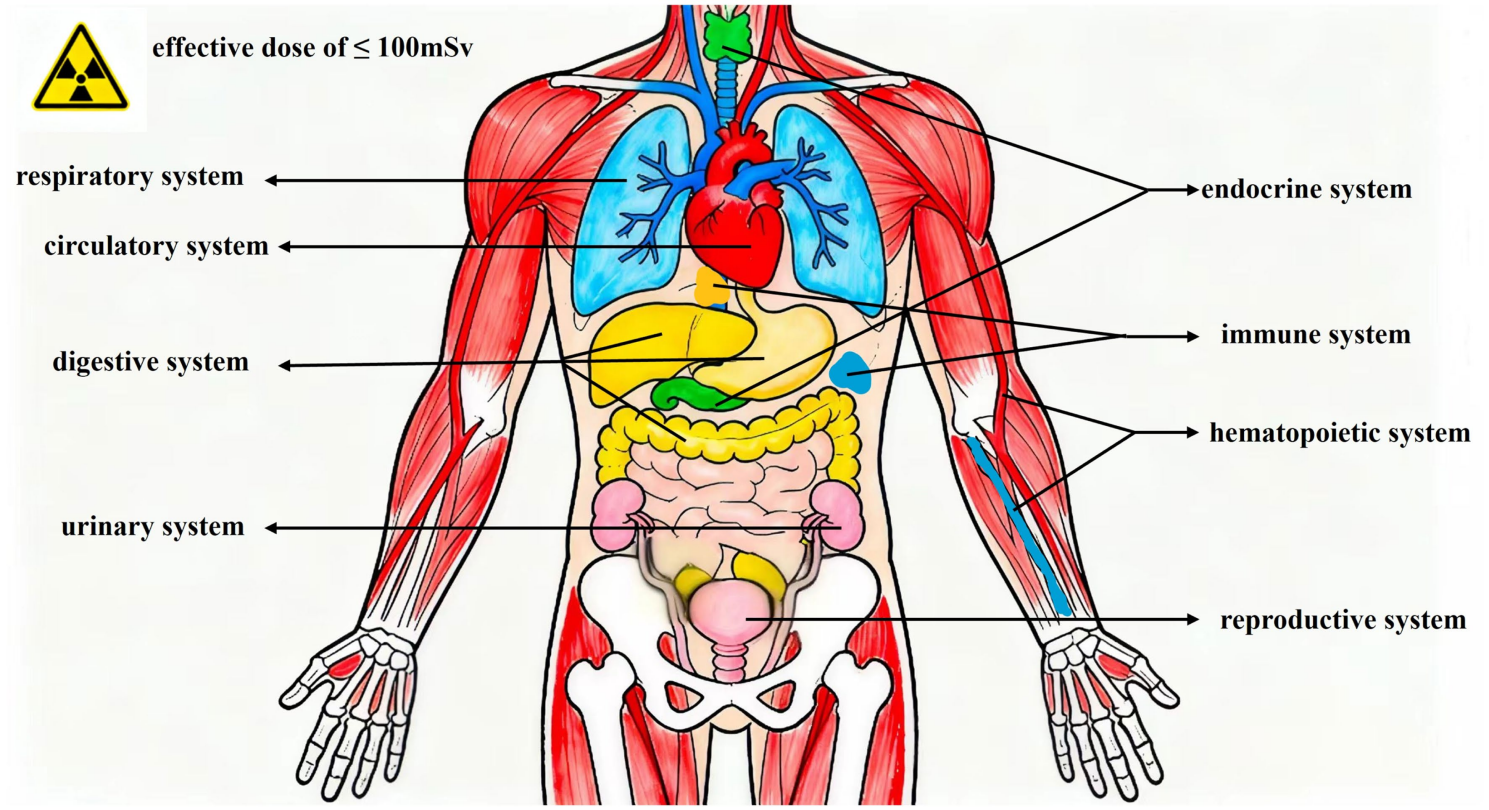
The effects of long-term LDIR on human health.

Although the majority of medical radiologists are exposed to radiation doses within the standard range, there is some degree of damage to human health from long-term, LDIR (9). It is, therefore, necessary to identify other early effects, considering cost-effectiveness, to avoid causing radiation damage to the health of medical radiologists. This paper describes the effects of long-term LDIR on human health, primarily involving impacts on the human Immune system, hematopoietic system, endocrine system, circulatory system, digestive system, reproductive system, respiratory system, and urinary system (Figure 1).

### 3.1 Effects of LDIR on the immune system

The human immune system is a complex defense mechanism evolved to protect the body against pathogens (e.g., bacteria, viruses, fungi, and parasites) and abnormal cells (e.g., cancer cells). Composed of numerous organs, cells, and molecules, the immune system is divided into two primary components: the innate immune system and the adaptive immune system. Based on their functions, immune organs are categorized into central immune organs and peripheral immune organs. Central immune organs, including the bone marrow and thymus, serve as critical sites for the production, differentiation, and maturation of lymphocytes. Peripheral immune organs, mainly consisting of lymph nodes, the spleen, and mucosal-associated lymphoid tissues, provide a platform for mature lymphocytes to recognize antigens and initiate specific immune responses. All these components work synergistically to maintain immune homeostasis and the body’s ability to resist infections.

Current epidemiological studies indicate that LDIR exerts a dual effect on the immune system, potentially causing immune impairment while, in certain cases, enhancing immune responses (9).

#### 3.1.1 Reduce the function of the immune system

Prolonged or sustained exposure to LDIR may lead to immunosuppression and increase the risk of infection and cancer. LDIR may affect the body’s immune response by inducing genetic mutations in immune cells, decreasing lymphocyte activity, affecting T and B cell function, and even impairing the hematopoietic system. Such effects may be particularly pronounced in populations with a history of long-term exposure (e.g., occupationally exposed persons). Epidemiological studies have focused on nuclear accidents, radiation therapy, and occupationally exposed persons. For example, nuclear power plant workers, radiologists, and nuclear medicine technicians who are chronically exposed to LDIR tend to show a higher risk of immune system damage. Long-term LDIR can damage hematopoietic stem cells, leading to a decrease in hematopoiesis, which in turn affects the production of immune cells and immune function.

The thymus, the primary site for the development, differentiation, and maturation of T lymphocytes, is highly sensitive to ionizing radiation. Ito et al. examined unique thymic tissues obtained from 165 individuals exposed to the Hiroshima atomic bomb explosion. Using bright-field immunohistochemistry and dual-color immunofluorescence, they conducted a detailed morphometric analysis of thymic activity and structure in these subjects at the time of their natural death, with comparisons to a separate cohort of unexposed control individuals. The results revealed increased histological markers of thymic involution in individuals exposed to LDIR compared to unirradiated counterparts. Although the atomic bomb radiation dose measurement method defined LDIR as 5–200 mGy (with doses exceeding 100 mGy also included in this range), this study still serves as a crucial reminder that LDIR may accelerate thymic degeneration (10). A 10-year investigation of the immune status of staff at a nuclear power plant found that the cumulative radiation dose among the staff ranged from 0.06 to 766.36 mSv. The results showed significant alterations in CD3^+^CD4^+^ helper-inducer cells—particularly their CD4^+^CD62L^+^ subset—as well as regulatory CD4^+^CD25^+^ cells and CD8^+^CD28^+^ cytotoxic subsets (11).

The spleen, as the largest peripheral immune organ, is the primary site of T and B lymphocyte activation. The spleen is a highly radiosensitive organ, and immune cells are considered to be among the most highly radiosensitive cells. The research has found that the effects of LDIR on the spleen are mainly manifested at the microscopic level. By comparing the peripheral blood lymphocyte, complement, and total immunoglobulin (IgG, IgA, IgM) subgroup levels of 50 radiology workers occupationally exposed to low levels of ionizing radiation and 35 age-matched healthy controls. Workers exposed to low levels of ionizing radiation were found to have weaker levels of CD4^+^ T lymphocytes and humoral immune responses (total immunoglobulin and complement) (12).

Dendritic cells (DCs), as specialized antigen-presenting cells of the innate immune system, play a pivotal role in the induction phase of adaptive immune responses. LDIR can hinder the maturation of DCs and downregulate the expression of their co-stimulatory molecules (e.g., CD80/CD86). Consequently, while DCs remain viable and capable of antigen contact, their functions become tolerant or incompletely activated. When these functionally impaired DCs interact with T cells, the lack of adequate co-stimulatory signals prevents the effective activation of naive T cells. Instead, this interaction may induce clonal anergy or immune tolerance in T cells, or even promote the differentiation of regulatory T cells (Treg). Ultimately, this disrupts immune homeostasis and increases the long-term risk of autoimmune diseases or a decline in immune surveillance function. DCs are responsible for antigen uptake, processing, and presentation, which can lead to either immune tolerance or activation. By disrupting the “initiator” of the immune response—the immune synapse between DCs and T cells—LDIR induces immune system dysfunction (13).

Long-term LDIR may trigger genetic mutations in immune cells, leading to immune cell dysfunction and even the induction of apoptosis or autoimmune responses. LDIR may induce apoptosis or autophagy in immune cells, especially in dendritic cells, macrophages, and T cells, which have strong immune surveillance functions and may induce their programmed death by triggering oxidative stress or DNA damage (14). Ionizing radiation can cause single or double-stranded DNA breaks in immune cells, and although cells usually repair them through repair mechanisms, incomplete or faulty repair may lead to mutations or even induce immune tolerance or immune escape. Radiation can cause cells to stay in the G1/G2 phase for DNA repair. LDIR may contribute to the development of tolerance in the immune system, resulting in a reduced immune response to certain pathogens or tumor antigens (15).

#### 3.1.2 Enhance the function of the immune system

Emerging studies have shown that LDIR can activate the immune system to a certain extent. Low levels of radiation can act as an “immune stimulant,” enhancing specific immune responses. For example, several studies have demonstrated that LDIR can stimulate the functions of DCs and macrophages, improving their ability to recognize and respond to foreign pathogens (16). This effect may exert a positive impact on immune surveillance in certain chronic diseases or tumors. It has been observed that LDIR exposure (e.g., following radiotherapy) can promote anti-tumor immune responses. Low doses of “sublethal” radiation (e.g., low-dose irradiation in radiotherapy regimens) may induce local immune responses, which are sometimes beneficial for cancer treatment but may also trigger immunosuppression in certain cases (17). The effects of long-term LDIR on the immune system are bi-directional, with the possibility of triggering either immunosuppression or activation of the immune response, depending on the dose of radiation, the duration and frequency of exposure, and the genetic and environmental background of the individual (18).

In contrast to its immunosuppressive effects, several studies have found that under specific conditions, LDIR exposure can stimulate the immune system, resulting in enhanced immune responses. The intercellular mechanism underlying the immune-stimulatory effect of LDIR primarily involves interactions between antigen-presenting cells and lymphocytes, with cell surface co-stimulatory molecules and secreted cytokines playing pivotal roles. LDIR can activate dendritic cells, macrophages, and other immune cells and improve their ability to identify and phagocytose pathogens. LDIR not only regulates the tumor microenvironment but also enhances the infiltration of activated T cells and initiates a series of inflammatory processes. In this case, the purpose of radiation is not to directly kill tumor cells, but to reprogram the immune system. Long-term LDIR may promote the body’s immune surveillance and enhance the clearance of tumor cells. LDIR can activate the body’s anti-tumor immune response, especially in radiotherapy. Radiotherapy eliminates tumor cells by localized high-dose radiation, while possibly further eliminating cancer cells not directly irradiated by activating the local immune response (19).

The formation of immune memory is an important mechanism by which the immune system responds to disease. Radiation may interfere with this memory process, in particular with the formation and maintenance of long-term immune memory, thereby affecting the body’s ability to respond to secondary infections. LDIR often affects the immune system through the production of free radicals and reactive oxygen species (ROS), and oxidative stress may cause damage to immune cells, altering their function and thereby affecting the immune response. In some cases, ROS can promote the activation of immune cells and enhance the immune response. For example, macrophages and dendritic cells can enhance their antigen-presenting function by releasing ROS. Adaptive response or IR excitatory effect induced by pre-exposure to LDIR. It has been reported that LDIR, known as “starter doses,” is administered before high-dose radiotherapy to minimize the side effects of high-dose radiation, known as “challenge doses.” Inhibition of TGF-*β* signaling during radiotherapy reduces the deleterious effects of radiation on normal tissues, and the adaptive response may involve the transcription of several genes and the activation of many protectives signaling pathways. Low-dose irradiation can induce protective mechanisms (e.g., *in vivo*) and reduce direct and indirect DNA damage (20).

Pre-exposure to LDIR induced up-regulation of anti-inflammatory and phagocytosis-related gene expression and down-regulation of key pro-inflammatory cytokine expression. It has potential neuroprotective effects on stimulating cell growth (21). LDIR also induces the production of anti-inflammatory cytokines by macrophages, which are involved in the change from an inflammatory to an anti-inflammatory microenvironment (22). Studies have shown that low-dose X-ray irradiation ranging from 0.3 to 0.7 Gy induces the relative maximum production of TGF-*β* through stimulated EC. This leads to a decrease in leukocyte/PBMC adhesion and may contribute to the anti-inflammatory effect of LD-RT (23). Using *in vivo* and *in vitro* experiments, Franz Rodel et al. showed that LDIT had anti-inflammatory effects in vitro and in vivo, affecting different cellular components and inflammatory mechanisms. Regulating the adhesion of PBMC to endothelial cells and its effect on activated macrophages may mediate the anti-inflammatory properties of LD-RT (24).

### 3.2 Effect of LDIR on the hematopoietic system

The hematopoietic system is highly sensitive to ionizing radiation, and alterations in peripheral blood parameters are an early indicator of radiation-induced organismal damage. Prolonged LDIR exposure causes bone marrow suppression and hematopoietic dysfunction, which in turn reduces peripheral blood cell counts. Additionally, LDIR increases the risk of hematological diseases and induces both morphological and quantitative changes in blood cells. These blood cell abnormalities can lead to a range of conditions, including reduced immunity, infections, inflammation, anemia, coagulation disorders, leukemia, myelodysplastic syndromes, and hemophagocytic syndromes (25). Dysfunction of the hematopoietic system caused by ionizing radiation is due to the ability of radiation to inhibit and destroy the normal functioning of hematopoietic stem cells, hematopoietic progenitor cells, and bone marrow naïve cells with the ability to proliferate, resulting in damage to the bone marrow sinusoids, as well as a decrease in peripheral blood leukocytes, hemoglobin, and platelets. It is noteworthy that changes in peripheral hematological parameters occur before the appearance of diseases of the hematopoietic system. Therefore, blood cell counts can be used to assess the degree of radiation impact on the hematopoietic system of radiation workers and can be used as a biological indicator for evaluating radiation hazards, and the level of peripheral hematological parameters can be used not only to evaluate the degree of damage to the hematopoietic system caused by ionizing radiation but also to assess the biological effects of radiation in the early stages, which has attracted the general attention of scholars both at home and abroad.

A prospective cohort study investigating platelet parameters in 1265 followed-up medical staff found that platelet parameters exhibited a trend of initial increase followed by a decrease. Furthermore, a dose–response relationship was observed between cumulative radiation dose and platelet changes (26). Employing the same prospective cohort study design, researchers collected health examination reports and personal dose monitoring data from 705 industrial radiation workers who underwent regular physical examinations at the Sixth People’s Hospital of Dongguan. The results revealed that red blood cell counts displayed a pattern of initial decrease, subsequent increase, and then a further decrease with increasing ionizing radiation exposure. This was contrary to the curve of platelet total count after irradiation. When the radiation dose was 2.904 mSv, the non-linear curve of hemoglobin count change reached a turning point. This indicates that long-term low-dose ionizing radiation has an impact on the blood cell levels of industrial radiation workers. There is a non-linear dose–response relationship between the red blood cell, platelet, and hemoglobin counts and the cumulative radiation dose (27). Another study investigated different parameters of the peripheral blood of 124 men who worked or lived in a radioactive contaminated environment after the Chernobyl accident. The results showed an increase in common signs of inflammatory response. For example, the increase in the number of white blood cells and the absolute number of white blood cells, the disproportionately high number of monocytes and band-shaped neutrophils, and the increase in plasma cytokine levels (28). It was also found that gender and working age had a certain degree of influence on the peripheral blood picture, which shows that the peripheral blood picture of occupational groups exposed to long-term LDIR may be affected by a variety of factors, including gender, age, working age, type of work, grade of the medical unit, smoking habits, family history of cancer, and so on.

### 3.3 Effects of LDIR on the endocrine system

The endocrine system is a fluid-based regulatory system consisting of various endocrine glands and scattered endocrine cells throughout the body. It synthesizes and secretes highly potent hormones that directly enter the bloodstream and act on distant target organs or cells, enabling precise regulation of bodily growth and development, metabolism, internal environmental homeostasis, and reproductive functions. Its key component organs include classic endocrine glands primarily responsible for hormone secretion, such as the hypothalamus (the neuroendocrine integration center), the pituitary gland (the master gland), the thyroid gland, the parathyroid glands, and the adrenal glands. These glands are involved in local and systemic regulation of blood pressure, red blood cell production, and digestive processes. This system maintains the coordination of body functions and internal environment stability through hierarchical regulation and negative feedback mechanisms.

The thyroid gland is the largest endocrine gland in the human body and the largest endocrine gland in adults. It is an endocrine organ that is controlled by the hypothalamic–pituitary-thyroid axis, which maintains the regulation of thyroid hormones. LDIR may produce functional or morphological changes that affect hormone secretion. Radiation dose is associated with the incidence of thyroid diseases such as hyperthyroidism, hypothyroidism, nodules, and carcinogenesis. A retrospective cohort study involving 585 hospital staff in Rome found that exposed workers had an increased risk of thyroid abnormalities, particularly hypothyroidism and goiter. Gender and age emerged as key risk factors for thyroid disorders, with women and older individuals demonstrating greater susceptibility (29). LDIR disrupts the levels of thyroxine (T4), triiodothyronine (T3), thyrotropin (TSH), free triiodothyronine (fT3), and free thyroxine (fT4), thereby inducing thyroid damage in radiation workers. A 12-year follow-up study of 326 workers revealed that T3 and T4 levels decreased, while TSH did not. Additionally, a negative dose–response relationship was observed between the duration of exposure and the decline in T3 serum levels (30). Cioffi DL et al. divided 120 medical staff exposed to ionizing radiation into three groups: 60 with an annual potential exposure dose of 6–20 mSv and 60 with an annual potential exposure dose of 1–6 mSv. These groups were compared with 60 unexposed medical staff (the control group). The results showed that the serum TSH levels of the exposed group were higher than those of the non-exposed group, while the fT3 and fT4 levels were lower than those of the non-exposed group. This further indicates that low-dose exposure to ionizing radiation can significantly affect the fT3, fT4, and TSH levels of medical staff, increasing the risk of thyroid dysfunction (hypothyroidism) in medical staff (31).

Most hormones play a role after being secreted into the blood by organs. Therefore, the effect of LDIR on blood hormone levels is also worthy of attention. Girinsky’s research demonstrated that whole-body irradiation at doses of 1.35 and 2 mGy induced a slight and transient increase in blood ACTH levels without any detectable rise in cytokine concentrations. In contrast, 10 Gy total body irradiation triggered marked elevations in plasma IL-6 and TNF levels, accompanied by activation of the hypothalamic–pituitary–adrenal axis. However, the precise mechanisms through which ionizing radiation induces cytokine production—including gene expression and the signal transduction pathways leading to gene transcription—remain to be fully elucidated (32).

### 3.4 Effects of LDIR on the circulatory system

Cardiovascular diseases are common disorders of the circulatory system, such as heart failure and coronary artery disease. The range is wide and the clinical manifestations vary. The clinical manifestations of radiation-induced cardiovascular disease depend on a variety of factors, such as dose, dose rate, volume of the exposed heart, age of exposure, disease latency, duration of follow-up, and other confounding factors (e.g., smoking and diet) (33). Ionizing radiation causes pericarditis, congestive heart failure, and coronary artery disease, and may also cause valve disease, arrhythmias, and conduction abnormalities (34). The ICRP report states that after 10 years of exposure to LDIR, approximately 1% of exposed individuals will develop cardiovascular and cerebrovascular disease (35).

Suzuki K analyzed the function of the cardiovascular system by collecting the blood pressure and heart rate changes of 3,104 radiation workers, using electrocardiogram changes as indicators, showed that blood pressure abnormalities among radiation workers tended to increase with age and length of service, and were higher among men than among women, a result which is consistent with the situation in the general population (36). Other studies have shown that workers exposed to external gamma radiation at doses higher than 100 mGy have a significantly increased risk of cardiovascular disease compared to workers exposed to lower doses (37).

### 3.5 Effects of LDIR on the digestive system

The liver is one of the main metabolic organs in the human digestive system, with functions such as bile secretion, metabolism, and detoxification etc. The liver is more sensitive to radiation. LDIR will produce certain radiation damage effects on the organism of practitioner, and the liver is also one of the sensitive organs to radiation. Some studies have shown that LDIR causes damage to liver cells and liver tissues, resulting in abnormal liver function. Long-term LDIR can lead to liver damage caused by ultrastructural changes in hepatocytes and liver enzymes, of which ALT and GGT are important indicators of liver damage.

By studying the liver and kidney functions of radiation workers exposed to LDIR, the results showed that the levels of alanine aminotransferase (ALT), urea nitrogen (BUN) and creatinine (CR) were higher than those of the control group, which further suggests that long-term, low-dose exposure to ionizing radiation has a certain degree of damage to the liver and kidney functions of radiation workers. Multivariate logistic regression analysis showed that the average annual effective dose was a risk factor for abnormal ALT and GGT in radiation workers. The results indicate that LDIR can cause some abnormal liver function in radiation workers, and low-dose ionizing radiation is a risk factor for abnormal ALT and GGT in radiation workers (38).

Chemotherapy and radiotherapy can cause significant changes in the composition of the intestinal microbiota, and these changes may also be related to the formation of mucositis (39). Studies have shown that LDIR can alter the intestinal microbiota community, and the changes in bacterial quantity are positively correlated with the radiation dose. The microbial metabolic axis plays a key role in generating a wide range of radiation protection effects and provides promising therapeutic targets for treating the adverse side effects of radiation exposure (40). The impact of LDIR on the human intestinal microbiota reveals a subtle ecological regulatory relationship that goes beyond traditional understanding. It is regarded as a stressor that can interfere with and possibly guide the microbiota toward a more adaptable and beneficial evolutionary state (41).

### 3.6 Effects of LDIR on the reproductive system

LDIR exerts adverse effects on male reproductive health, primarily by disrupting testicular structure and sperm production. Histological investigations have shown that LDIR reduces testis weight and sperm count, impairs spermatogenesis, and induces degenerative changes in the seminiferous tubules (42). The testis plays a critical role in balancing immune defense against infections and sustaining spermatogenesis—a delicate equilibrium highly vulnerable to disruption by LDIR exposure, which may lead to long-term reproductive health consequences (43).

The ovary is the most radiation-sensitive component of the female reproductive system. Unlike the male testes, which continuously generate sperm, female ovaries contain a fixed number of primary follicles (approximately 100–200 million) at birth, with this number gradually declining with age. Radiation directly damages these valuable, non-renewable follicles. Martino et al. used cumulus-oocyte complexes (COCs) isolated from the ovaries of juvenile sheep and human ovaries as complementary *in vitro* models to investigate the effects of low-dose X-ray exposure (used in medical diagnostics) on female fertility (44). The study found that the minimum dose of 10 mGy had no significant impact on most measured parameters at the oocyte and embryo levels. In contrast, 50 and 100 mGy X-ray exposure reduced oocyte bioenergetic/oxidative activity, though no visible effects on oocyte or embryo development were observed. Additionally, blastocyst bioenergetic/oxidative status was reduced across all tested doses.

In the context of reproduction and genetics, LDIR can cause damage to the health of occupationally exposed populations, with most genotoxic effects being severe, irreversible, and irreparable (45). Ionizing radiation damages the DNA of immune cells either directly or indirectly; while cells can repair some of this damage via DNA repair mechanisms, incomplete or erroneous repair may result in mutations or apoptosis. Radiation-induced DNA damage in peripheral blood lymphocytes is predominantly characterized by complexity and clustering—key features of such damage. When DNA breaks occur, the formation of DNA adducts inhibits DNA repair, leading to chromosomal aberrations or the appearance of micronuclei, which in turn increase cellular radiosensitivity (46). Consequently, chromosomal aberrations and micronuclei in human peripheral blood are recognized as specific biomarkers of the biological effects of long-term LDIR exposure, and hold significant value for diagnosing radiation-induced health damage in occupationally exposed groups. Among radiation workers, chromosomal aberrations and micronuclei in peripheral blood lymphocytes are the most widely used cytogenetic indicators for assessing radiation damage caused by long-term LDIR exposure (47). Chromosomal aberrations directly reflect the extent of chromosomal damage, while micronuclei are considered to be entire chromosomes or chromosome fragments that are broken or lost during mitosis. Studies have confirmed that the number of micronuclei directly reflects the degree of chromosomal damage and indirectly indicates the extent of radiation-induced organismal damage. Numerous investigations have shown that the frequency of chromosomal aberrations and micronuclei in peripheral blood lymphocytes of radiation workers is significantly higher than in the general population (48).

Radiation workers—particularly those engaged in interventional procedures—represent the primary group exposed to long-term LDIR. Their elevated risk of chromosomal aberrations and long-term cancer risk warrants substantial attention. Eken et al. compared 40 radiology staff members with occupational radiation exposure to 30 control subjects and found a significant increase in micronucleus (MN) frequency in the exposed group (49). This study confirmed that the rate of chromosomal aberrations in peripheral blood lymphocytes of radiation workers is significantly higher than in the control group, with a higher chromosomal aberration rate correlating with an increased future cancer risk. Notably, a clear positive correlation was observed between the chromosomal aberration rate and cumulative radiation dose.

### 3.7 Effects of LDIR on the respiratory system

Chest X-ray examinations and low-dose chest computed tomography (CT) are currently the most widely used clinical methods for lung cancer screening; however, cumulative ionizing radiation exposure from these procedures can induce or exacerbate cancer.

A recent retrospective study evaluated 5,203 participants (3,439 men and 1,764 women) who underwent 42,228 low-dose CT scans and 635 positron emission tomography-computed tomography (PET-CT) scans over a 10-year period (50). The median cumulative effective dose at the 10th year of screening was 9.3 mSv for men and 13.0 mSv for women. Stratified by age and sex, the lifetime attributable risk of lung cancer and major cancers following 10 years of CT screening ranged from 1.4 to 5.5 cases per 10,000 screened individuals and from 2.6 to 8.1 cases per 10,000 screened individuals, respectively. Specifically, women aged 50–54 had a lifetime attributable risk of lung cancer and major cancers that was approximately fourfold and threefold higher, respectively, than that of men aged 65 years and older.

Despite the radiation exposure and associated cancer risk inherent in low-dose CT lung cancer screening, these risks are considered acceptable given that such screening increases lung cancer detection rates and significantly reduces lung cancer mortality.

### 3.8 Effects of LDIR on the urinary system

At present, there are few studies that have investigated the direct impact of LDIR on the urinary system. However, in some cancer-related studies, it has been shown that the cumulative effect of LDIR increases the incidence of tumors in the skeletal muscle system and urinary system.

Based on an updated analysis of the International Nuclear Workers Study (INWORKS) involving 309,932 nuclear industry workers from three countries (the United Kingdom, France, and the United States), the study found a significant association between cumulative radiation dose and the mortality rate of urinary system cancers after long-term exposure to low-dose ionizing radiation. Specific data showed that for every 1 gray (Gy) increase in cumulative radiation dose, the relative risk of dying from urinary system cancer increased significantly by 186% (excess relative risk ERR/Gy = 1.86). This result indicates that in the context of occupational exposure, the urinary organs may be one of the sensitive targets for carcinogenesis by low-dose ionizing radiation (51).

### 3.9 Other effects of LDIR

In frontier research, the effects of ionizing radiation on brain cells have been extensively investigated. Researchers have used brain organoids derived from human induced pluripotent stem cells to study the radiosensitivity of human brain cells, with studies showing that acute LDIR exposure can cause subclinical cellular damage, leading to altered gene expression and cellular function in the human brain (52). Brain organoids serve as a valuable tool for monitoring acute changes in cellular status and function following ionizing radiation exposure and hold potential for long-term tracking of these effects. Changes in brain cell function also impact memory: a study comparing neuropsychological test scores between 83 interventional cardiologists and 83 control subjects found that the interventional cardiologists exhibited significant memory decline, primarily affecting verbal long-term memory and verbal fluency (53).

The lens is one of the most radiation-sensitive tissues in the human body and the most sensitive tissue in the eye, with lens epithelial cells showing particular sensitivity to ionizing radiation. Cataract is the leading cause of visual impairment and blindness globally, accounting for 51% of all blindness cases across all eye diseases. China has the largest population of individuals with cataract-induced blindness. Among reported radiation-related diseases, cataract ranks among the top three, representing 16.04% of all such cases. Cataract is characterized by the progressive opacification of the eye lens, which ultimately leads to visual impairment. Importantly, radiation-induced lens damage is relatively common among medical staff with long-term radiation exposure. Current epidemiological studies confirm that long-term ionizing radiation exposure can induce cataract (54). LDIR causes DNA breaks in lens epithelial cells through direct or indirect damage; unrepaired DNA damage may alter cell proliferation, exert adverse effects on cell differentiation, and induce changes in lens morphology.

A comprehensive assessment—including visual acuity testing, slit-lamp examination of the crystalline lens, and evaluation of the vitreous, fundus, and visual fields—in radiation workers found that the primary sites of crystalline lens abnormalities were punctate and cortical opacities in the equatorial region of the lens, followed by reduced lens transparency and posterior capsule opacification (55). In conclusion, the effects of long-term low-dose ionizing radiation exposure on the eye lens of medical professionals are associated with genetic factors, sex, age, job type, and length of service.

### 3.10 Effects of constant LDIR on the human health system

Notably, beyond occupational workers exposed to nuclear industry and medical radioactive sources, individuals in numerous specific regions worldwide are exposed to ionizing radiation via natural sources. Examples of such regions include Ramsar (Iran), Kerala (India), and Yangjiang (China)—collectively referred to as high natural background radiation areas (HBRAs) (8). Natural background radiation comprises cosmic rays (neutrons and other particles), terrestrial radioactivity (daughters of uranium and thorium, including radon gas), and radioactivity from biological sources (notably potassium-40 and carbon-14). Studies on populations residing in HBRAs (e.g., Yangjiang in Guangdong Province, China; Kerala, India; and Ramsar, Iran) have provided valuable natural observation models. These studies reveal that despite lifelong exposure to high background radiation, the health effects on local populations are complex: some individuals may undergo “pre-adaptation” of their cellular defense systems through intergenerational or lifelong exposure, resulting in enhanced genomic stability and more robust DNA damage response capabilities. This phenomenon directs attention to the molecular basis of individual differences: polymorphisms in DNA repair genes. The annual dose rates experienced by residents in these regions range from approximately 1.51–80 mSv/year in Ramsar (Iran), 1.51–45 mSv/year in Kerala (India), and 1.5–5.44 mSv/year in Yangjiang (China).

Ramachandran et al. (56), Das et al. (57), and Karupassamy et al. (58) focused on chromosomal aberrations and micronuclei as endpoints but found no significant differences in the levels of these markers between populations in HBRAs and normal natural background radiation areas (NHBRAs). When DNA damage was assessed as an endpoint, all studies showed that cells derived from HBRA residents had either similar or lower levels of DNA strand breaks compared to cells from NHBRA residents. The vast majority of reviewed studies also failed to detect a significant difference in cancer incidence between HBRA and NHBRA cohorts (59)—a finding that contradicts many hypotheses regarding ionizing radiation-induced carcinogenesis. When immune system biomarkers were targeted, for instance, Li et al. examined immune cell populations and inflammatory biomarkers and found no differences in cell subtype distribution between HBRA and NHBRA groups. However, five inflammatory markers (IFN-*γ*, MCP-1, sIL-6R, EGFR, and CRP) were significantly upregulated with increasing cumulative radiation dose (60).

Many findings from studies on the health effects of HBRAs differ from those observed in occupationally exposed populations. These discrepancies highlight the need for careful analysis of conclusions by refining factors such as radiation dose, absorbed dose, and age.

## 4 Discussion

Existing studies on the health effects of long-term LDIR have laid a foundational groundwork for understanding its systemic impacts. However, significant variations in research quality and inherent limitations necessitate critical scrutiny. Regarding strengths, most studies employ rigorous cohort designs and standardized dose-monitoring methods, ensuring the reliable quantification of cumulative radiation exposure. Furthermore, the integration of multi-dimensional endpoints—including hematological parameters, hormone levels, cytogenetic markers (chromosomal aberrations and micronuclei), and clinical outcomes—enhances the comprehensiveness of effect assessment. However, notable limitations persist. First, population selection bias is prevalent: most studies focus on occupational groups (e.g., radiologists, nuclear power plant workers) with relatively homogeneous exposure scenarios, while data on general populations (e.g., individuals exposed to environmental radiation) remain scarce. Second, confounding factor control is inadequate: factors such as age, gender, smoking habits, and underlying diseases are often not fully adjusted, leading to potential overestimation or underestimation of LDIR’s effects (e.g., gender differences in cardiovascular risk and thyroid sensitivity were only partially addressed). Third, long-term follow-up is lacking: most studies have a follow-up duration of 10–15 years, failing to capture delayed effects (e.g., late-onset cancer, chronic organ dysfunction) that may manifest decades after exposure. Fourth, methodological inconsistencies in dose calculation (e.g., variations in defining “low dose” across studies) hinder cross-study comparisons and analyses.

The literature presents contradictory findings that reflect the complexity of LDIR’s biological effects, with two core contradictions being particularly prominent. The first contradiction pertains to the dual effects of LDIR on the immune system: while most studies confirm its immunosuppressive effects (e.g., reduced CD4 + T lymphocyte counts, impaired DC maturation), others report immune activation (e.g., enhanced anti-tumor immunity, increased macrophage phagocytic activity). This discrepancy arises from three key factors: (1) Dose and dose rate: low doses (≤0.5 Gy) tend to induce adaptive immune responses, whereas prolonged exposure to higher cumulative doses (≥50 mSv) is more likely to cause immunosuppression; (2) exposure context: therapeutic LDIR (e.g., adjuvant radiotherapy) may induce local anti-tumor immunity, whereas occupational continuous exposure is more likely to cause systemic immunosuppression; and (3) individual heterogeneity: genetic background (e.g., DNA repair gene polymorphisms) determines the threshold for immune activation or suppression.

The second contradiction involves health outcomes in high natural background radiation areas (HBRA): unlike occupational exposure populations, HBRA inhabitants (e.g., in Yangjiang, China; Ramsar, Iran) show no significant increase in cancer incidence or cytogenetic damage, despite long-term exposure to doses (1.5–80 mSv/year) exceeding normal background levels. This inconsistency challenges the linear-no-threshold (LNT) model and suggests that adaptive responses (e.g., enhanced DNA repair capacity, pre-activated cellular defense systems) may mitigate potential harm in HBRA populations. In contrast, occupational exposure is often characterized by intermittent high-dose “spikes” (e.g., during interventional procedures) combined with chronic low-dose accumulation, which may overwhelm adaptive mechanisms and induce cumulative damage. Despite substantial progress, critical knowledge gaps remain that limit the development of precise radiation protection strategies and risk assessment frameworks. First, lack of research on multi-system interactive effects: Current studies focus on single organs/systems (e.g., thyroid, hematopoietic system) but ignore the interconnectedness of the human body. For example, LDIR-induced gut microbiota alterations may indirectly affect immune function and endocrine regulation, yet few studies explore such cross-system crosstalk. Second, inadequate data on vulnerable populations: Children, pregnant women, and individuals with chronic diseases are understudied, leaving their specific risks and protective needs unaddressed.

The findings of this review align with and expand the conceptual framework proposed earlier, particularly the dualistic model of deterministic and stochastic effects by the International Commission on Radiological Protection (ICRP) and the radiation adaptive response theory. For deterministic effects, existing data strongly support the dose-threshold relationship: LDIR rarely induces acute tissue damage (e.g., cataracts, hematopoietic suppression) but causes subclinical, cumulative changes (e.g., thyroid hormone imbalance, liver enzyme abnormalities) that may progress to clinical disease with prolonged exposure. This confirms that deterministic effects are not exclusive to high doses but manifest as chronic, low-grade damage in LDIR scenarios. For stochastic effects, the LNT model is partially challenged by HBRA studies, which suggest that adaptive responses may limit cancer risk at low doses. However, occupational studies consistently show increased risks of hematological malignancies and urinary system cancers, indicating that the LNT model remains relevant for cumulative occupational exposure—likely due to the absence of pre-adaptive mechanisms in non-HBRA populations.

The radiation adaptive response (RAR) (or “priming dose effect”) serves as a key bridge between these observations: pre-exposure to low doses can activate protective pathways (e.g., DNA repair, antioxidant enzymes), explaining the resilience of HBRA populations. In contrast, occupational exposure often lacks such pre-adaptive conditioning, leading to stochastic effects. Additionally, DNA repair gene polymorphisms further refine the framework by explaining why individuals with the same exposure dose exhibit varying responses—highlighting the need for personalized risk assessment.

## 5 Summary and prospect

This review provides a comprehensive overview of the complex effects of low-dose ionizing radiation on various human body systems. Its effects cannot be oversimplified by the traditional binary classification of “harmful” or “non-harmful.” Notably, the bidirectional effects of LDIR on the immune system alone challenge conventional notions. Only through a thorough understanding of the deterministic and stochastic effects of ionizing radiation can the protective awareness of occupational workers be effectively enhanced. The current studies are all based on the cumulative effect of LDIR doses. This seems to support the deterministic effect more, while the stochastic effect is more evident in cancer research. Studies on the incidence of cancer in radiation workers caused by LDIR mainly focus on radiation-sensitive sites such as the thyroid gland, reproductive system, and bone marrow hematopoietic system. Ionizing radiation at any dose, under random effects, increases the risk of cancer. From deterministic effects analysis, at low dose levels, the risk of excess cancer is proportional to the cumulative radiation dose. This paper reviews research results at home and abroad on the effects of long-term exposure to LDIR on the human health system, explores the population health effects caused by it, analyzes the effects of long-term LDIR on various organs and systems of the human body, and provides a basis for research into the mechanism of long-term low-dose ionizing radiation-induced health effects on the organism and research into targeted protection strategies.

Radiation injury is an important public health problem that threatens the health of occupational radiologists, and occupational exposure to ionizing radiation is the most important occupational disease hazard to the health of radiological workers. At present, the effects of long-term LDIR on the human health system are a complex issue. Although studies on the effects of LDIR on the human immune system, hematopoietic system, and reproductive system have been conducted and some preliminary results have been obtained, such as the dual effects of long-term LDIR on immune cells, and the damage to DNA and chromosomes, there is still a large gap in the research field regarding the combined effects of multiple systems. Research on the health effects of ionizing radiation still faces a series of challenges; no consistent conclusions have been drawn on some of the health effects, and the risk assessment of ionizing radiation is at a stage of continuous development and refinement, particularly regarding the health effects of low-dose ionizing radiation. However, further research and exploration are still needed on how to avoid the adverse effects of radiation on the immune system while ensuring the safety and effectiveness of radiation applications.

The current research focus is on the effects of low-dose ionizing radiation on a specific organ or system, and it has delved into the molecular and cellular levels. However, the human health system is composed of many complex, precise, and interrelated mechanisms. For example, in the impact of low-dose ionizing radiation on the immune system, it not only involves various immune cells, but is also closely related to DNA and chromosomes in the reproductive system. Therefore, when considering radiation damage and protection, all systems should be interconnected and comprehensively considered. Most studies show that the cumulative radiation dose is the key to triggering occupational diseases, which is the manifestation of the deterministic effect. Therefore, in terms of occupational protection, strengthening the monitoring of individual doses of radiation for medical staff and assessing their health conditions is of great significance for determining the biological effects of radiation and the critical values of ionizing radiation doses. It provides a scientific basis for revising the basic standards of radiation protection and effectively protecting radiation injuries of medical staff.

In summary, long-term exposure to LDIR may damage many parts of the human body, including the human Immune system, hematopoietic system, endocrine system, circulatory system, digestive system, reproductive system, respiratory system, and urinary system, and the effects of low-dose exposure to ionizing radiation on human health should not be ignored (Table 1). The differences in the findings of the studies may be related to the differences in the selection of the population, sample size, cumulative exposure dose, and methods of analyzing the data. Future studies on long-term exposure to LDIR need to take full account of the influence of genetic background and other confounding factors to further explore their health effects. A comprehensive health monitoring mechanism should be created, the working environment should be continuously improved, and occupational health examinations and dose monitoring should be carried out regularly, to effectively reduce the dose received by radiological workers; radiological workers should effectively carry out occupational protection, strictly comply with operating procedures, and raise their awareness of self-protection. Future studies on long-term exposure to LDIR need to take full account of the influence of genetic background and other confounding factors to further explore their health effects. A comprehensive health monitoring mechanism should be created, the working environment should be continuously improved, and occupational health examinations and dose monitoring should be carried out regularly, to effectively reduce the dose received by radiological workers; radiological workers should effectively carry out occupational protection, strictly comply with operating procedures, and raise their awareness of self-protection.

**Table 1:** Summarize the effects of LDIR on the human health system.

Authors and publication year	Title	Region	Dose and duration	Effect
Ito R et al., 2017 (10)	Late Effects of Exposure to Ionizing Radiation and Age on Human Thymus Morphology and Function	Hiroshima	low (5–200 mGy) or moderate-to-high (>200 mGy) doses	Immune system
Gyuleva IM et al., 2017 (11)	Assessment of Some Immune Parameters in Occupationally Exposed Nuclear Power Plant Workers: Flow Cytometry Measurements of T Lymphocyte Subpopulations and Immunoglobulin Determination. Dose Response	Kozlo-duy	0.06 to 766.36 mSv	Immune system
Godekmerdan A et al., 2004 (12)	Diminished cellular and humoral immunity in workers occupationally exposed to low levels of ionizing radiation	Chernobyl	3.5 mSv	Immune system
Kiselev SM et al., 2017 (14)	IMMUNOLOGICAL MONITORING OF THE PERSONNEL AT RADIATION HAZARDOUS FACILITIES	Russia	25 to 100 mSv	Immune system
Leuraud K et al., 2015 (25)	Ionizing radiation and risk of death from leukemia and lymphoma in radiation-monitored workers (INWORKS): an international cohort study	USA	1.1 mGy	Hematopoietic system
Ning Liu et al., 2021 (26)	Effects of exposure to low-dose ionizing radiation on changing platelets: a prospective cohort study	Guangdong	50 mSv	Hematopoietic system
Jia-jia Guo et al., 2022 (27)	Dose–Response Effects of Low-Dose Ionizing Radiation on Blood Parameters in Industrial Irradiation Workers	Dongguan	2.904 mSv	Hematopoietic system
Cioffi DL et al., 2025 (31)	Low dose ionizing radiation exposure and risk of thyroid functional alterations in healthcare workers	Italy	6 and 20 mSv	Endocrine system
Azizova TV et al., 2014 (37)	Cerebrovascular diseases incidence and mortality in an extended Mayak Worker Cohort 1948–1982	Mayak Production Association	54 ± 76 mSv	Circulatory system
Mousavikia SN et al., 2023 (45)	Evaluation of micronuclei and antioxidant status in hospital radiation workers occupationally exposed to low-dose ionizing radiation	Iran	50 mGy	Reproductive system
Rampinelli C et al., 2023 (50)	Exposure to low dose computed tomography for lung cancer screening and risk of cancer: secondary analysis of trial data and risk–benefit analysis	Italy	9.3 mSv	Respiratory system
