# Supplementary material for: The impact of long-term low-dose ionizing radiation on human health: risks and protective measures
Source: Front Med (Lausanne). 2026 Jan 29;13:1628683. doi: 10.3389/fmed.2026.1628683 (PMC12893970; doi:10.3389/fmed.2026.1628683)
Supplement: Supplementary file 3 [file Supplementary_file_1.docx]

# ===============================================================

# Hass Avocado Adoption Models – Paper Figure Generator v5

# ===============================================================

# Genera todas las figuras del artículo en formato .SVG y .PDF

# Incluye etiquetas A, B, C… y las guarda en carpeta /images

# ===============================================================

# -----------------------------

# 1. Instalar y cargar paquetes

# -----------------------------

paquetes <- c("readxl", "dplyr", "broom", "purrr", "janitor", "tidyr", "corrplot",

"psych", "ggplot2", "pROC", "svglite", "sf", "tmap", "stringr", "forcats")

instalar <- paquetes[!paquetes %in% installed.packages()]

if (length(instalar) > 0) install.packages(instalar)

lapply(paquetes, library, character.only = TRUE)

# -----------------------------

# 2. Preparar entorno

# -----------------------------

dir.create("images", showWarnings = FALSE)

set.seed(123)

# -----------------------------

# 3. Leer y limpiar datos

# -----------------------------

df <- read_excel("base de datos aguacate 23.24.xlsx") %>% clean_names()

# Variables dependientes (outcomes)

variables_objetivo <- c("fertilizante_organico", "fertilizante_quimico", "riego", "analisis_suelos_1")

# Variables explicativas (predictors)

variables_predictoras <- c("areasembrada", "edadcultivo", "nutricion", "gestionagua",

"vive_predio", "distanciakm", "tipoadministracion")

# -----------------------------

# 4. Matriz de correlaciones

# -----------------------------

cor_matrix <- cor(df %>% select_if(is.numeric), use = "pairwise.complete.obs")

# Correlograma (Figura C)

svglite("images/Figure_C_correlogram.svg", width = 10, height = 10, bg = "white")

corrplot(cor_matrix, method = "circle", type = "upper",

col = colorRampPalette(c("red", "white", "blue"))(200),

tl.col = "black", tl.srt = 45, tl.cex = 0.8, title = "C. Correlation Matrix")

dev.off()

pdf("images/Figure_C_correlogram.pdf", width = 10, height = 10, bg = "white")

corrplot(cor_matrix, method = "circle", type = "upper",

col = colorRampPalette(c("red", "white", "blue"))(200),

tl.col = "black", tl.srt = 45, tl.cex = 0.8, title = "C. Correlation Matrix")

dev.off()

# -----------------------------

# 5. Modelos Logísticos

# -----------------------------

ajustar_modelo <- function(var_obj) {

formula <- as.formula(paste0(var_obj, " ~ ", paste(variables_predictoras, collapse = " + ")))

df_modelo <- df[, c(var_obj, variables_predictoras)] %>%

mutate(across(all_of(var_obj), as.numeric)) %>%

filter(if_all(everything(), ~ !is.na(.)))

modelo <- glm(formula, data = df_modelo, family = binomial)

roc_obj <- roc(df_modelo[[var_obj]], predict(modelo, type = "response"))

auc_val <- auc(roc_obj)

tidy(modelo) %>%

mutate(

objetivo = var_obj,

odds_ratio = exp(estimate),

ci_low = exp(estimate - 1.96 * std.error),

ci_high = exp(estimate + 1.96 * std.error),

auc = auc_val

) %>%

select(objetivo, term, estimate, std.error, p.value, odds_ratio, ci_low, ci_high, auc)

}

tabla_resultados <- map_dfr(variables_objetivo, ajustar_modelo)

# -----------------------------

# 6. Forest Plot (Figura D)

# -----------------------------

tabla_plot <- tabla_resultados %>%

filter(term != "(Intercept)") %>%

mutate(

objetivo = factor(objetivo,

levels = c("fertilizante_quimico", "riego", "fertilizante_organico", "analisis_suelos_1"),

labels = c("Chemical Fertilizer", "Irrigation", "Organic Fertilizer", "Soil Analysis")),

term = str_replace_all(term, "_", " "),

term = str_to_title(term)

)

p_forest <- ggplot(tabla_plot, aes(x = odds_ratio, y = fct_reorder(term, odds_ratio), color = objetivo)) +

geom_point(position = position_dodge(width = 0.7), size = 2) +

geom_errorbar(aes(xmin = ci_low, xmax = ci_high),

position = position_dodge(width = 0.7), width = 0.25) +

geom_vline(xintercept = 1, linetype = "dashed", color = "gray40") +

facet_wrap(~objetivo, scales = "free_y") +

scale_x_log10() +

labs(

title = "D. Odds Ratios (OR) and 95% Confidence Intervals",

subtitle = "Logistic regression results across adoption outcomes",

x = "Odds Ratio (log scale)", y = "Predictor variable"

) +

theme_minimal(base_size = 11) +

theme(legend.position = "none",

plot.title = element_text(face = "bold", size = 13, hjust = 0),

axis.text.y = element_text(size = 9),

strip.text = element_text(face = "bold", size = 11))

ggsave("images/Figure_D_forest_plot.svg", p_forest, width = 12, height = 8)

ggsave("images/Figure_D_forest_plot.pdf", p_forest, width = 12, height = 8)

# -----------------------------

# 7. Barras de Certificaciones (Figura B)

# -----------------------------

cert_vars <- c("gap", "globalgap", "organic", "export", "rainforest")

df_cert <- df %>% select(any_of(cert_vars)) %>% pivot_longer(everything())

df_cert_summary <- df_cert %>% group_by(name) %>% summarise(rate = mean(value, na.rm = TRUE))

p_barras <- ggplot(df_cert_summary, aes(x = reorder(name, rate), y = rate)) +

geom_col(fill = "#2E7D32") +

coord_flip() +

labs(title = "B. Certification Adoption Rates",

x = "Certification type", y = "Share of producers (%)") +

theme_minimal(base_size = 12)

ggsave("images/Figure_B_certifications.svg", p_barras, width = 8, height = 6)

ggsave("images/Figure_B_certifications.pdf", p_barras, width = 8, height = 6)

# -----------------------------

# 8. Mapas Departamentales (Figuras E–H)

# -----------------------------

# Requiere shapefile de departamentos (descargado desde DANE/IGAC)

colombia <- st_read("departamentos_colombia.shp") # debe contener campo "DEPARTAMENTO"

for (i in seq_along(variables_objetivo)) {

var <- variables_objetivo[i]

resultado <- tabla_resultados %>% filter(objetivo == var)

mapa <- colombia %>%

left_join(resultado, by = c("DEPARTAMENTO" = "term"))

fig_letter <- LETTERS[i + 4] # E, F, G, H

tmap_mode("plot")

mapa_plot <- tm_shape(mapa) +

tm_polygons("estimate", palette = "-RdYlGn", title = "Coefficient") +

tm_layout(main.title = paste0(fig_letter, ". Department-level Coefficients – ", str_to_title(var)),

main.title.size = 1, legend.text.size = 0.8)

tmap_save(mapa_plot, filename = paste0("images/Figure_", fig_letter, "_map_", var, ".pdf"), width = 7)

tmap_save(mapa_plot, filename = paste0("images/Figure_", fig_letter, "_map_", var, ".svg"), width = 7)

}

# -----------------------------

# 9. Mensaje de finalización

# -----------------------------

cat("\n✅ Análisis y generación de figuras completados.\n")

cat("📁 Figuras guardadas en carpeta: ./images/\n")

cat("🖼️ A–H: Correlograma, Forest Plot, Barras y Mapas regionales listos.\n")
